# The effects of subjective loss of control on risk-taking behavior: the mediating role of anger

**DOI:** 10.3389/fpsyg.2015.00774

**Published:** 2015-06-15

**Authors:** Birgit M. Beisswingert, Keshun Zhang, Thomas Goetz, Ping Fang, Urs Fischbacher

**Affiliations:** ^1^Department of Empirical Educational Research, University of KonstanzKonstanz, Germany; ^2^Department of Empirical Educational Research, Thurgau University of Teacher EducationKreuzlingen, Switzerland; ^3^Graduate School of Decision Sciences, University of KonstanzKonstanz, Germany; ^4^Department of Psychology, Capital Normal UniversityBeijing, China; ^5^Department of Economics, University of KonstanzKonstanz, Germany; ^6^Thurgau Institute of EconomicsKreuzlingen, Switzerland

**Keywords:** perceived control, anger, risk-taking, attribution, cross-cultural study

## Abstract

Based on the Appraisal Tendency Framework on the antecedents and consequences of emotions two experimental studies examined the relationship between externally caused loss of control experiences and risk-taking behavior, as well as the assumed mediation of this relationship by the emotion anger. An experimental paradigm for inducing externally caused and consequently externally attributed loss of control which should lead to experiences of anger was developed and pretested in a Pilot Study. The relationship between loss of control experiences, anger, and risk-taking behavior was investigated using two separate student samples from Germany (*N* = 84, 54% female) and China (*N* = 125; 64% female). In line with our hypotheses, results showed that anger mediated the link between subjective loss of control experiences and increasing risk-taking behavior. Multiple group comparisons revealing similar patterns in both samples affirmed the results’ cross-cultural generalizability. These results implicate that anger makes people less risk averse in the process of economic decision making.

## Introduction

“Change is the only constant.”

(Heraclitus of Ephesus, approximately 535–475 BC)

(Heraclitus, 2001)

The world keeps changing around us. Ongoing rapid development and new technologies continue to change our environments as well as our living and working conditions (Schober et al., 2007). People are facing new challenges every day as everyone has to quickly learn about the latest development, to newly orient, to adapt and get along with the new circumstances, and unknown environments. People might find these experiences demanding and associate them with a loss of orientation and subjective feelings of uncertainty and loss of control.

The question of how externally caused experiences of loss of control affect people’s decision-making processes bears wide practical relevance. In this study, we investigate the effects of experimentally administered subjective loss of control experiences on risk-related decision making. It seems plausible to assume that emotions play a special role in this relationship for two reasons. First, perceived control is known to be a cognitive antecedent of emotions such as anxiety or hopelessness (e.g., Skinner, 1996; Goetz et al., 2010), and, second, due to the well-known impact of emotional experiences on decision-making processes (e.g., Loewenstein and Lerner, 2003). This study focuses on the role of anger in particular as one central emotion and examines its influence on the relationship between loss of control experiences and risk-taking behavior. Additionally, the question arises as to whether the assumed functional associations are generalizable, especially across people from various cultures. Despite potential cultural mean level discrepancies in control perceptions and emotions (e.g., see Eid and Diener, 2001; Spector et al., 2004), differences in structural relations and functional mechanisms are not to be expected from a theoretical perspective as the herein investigated variables (control perceptions and emotions as well as their consequences) are considered to be basic and universal (e.g., see Heckhausen and Schulz, 1995; Skinner, 1996; Pekrun, 2006). We explore the cross-cultural generalization with samples from Germany and China representing different cultural backgrounds.

## Relation between Subjective Control and Risk-Taking Behavior

There is a close association between perceptions of control and risk taking. Although Skinner (2007, p. 912) considers risky situations to be “prototypical cases of uncontrollability,” subjective perceptions of control are an important facet of the perceived riskiness of a hazard or situation. For example, illusions of control (Langer, 1975; for a review, see Thompson et al., 1998) have been shown to play an important role in chance situations and, thus, in the evaluation of and attitudes toward risks. Perceived controllability of a hazard was identified as essential to one of two factors (i.e., “dread”) underlying people’s risk perceptions (Slovic, 1987; Peters and Slovic, 1996). Thus, it is reasonable to assume that perceived control impacts risk perception and risk-related behavior (Thaler and Johnson, 1990; Renn, 1998).

Perceived control is understood as the extent to which one believes that he or she can predict or influence events (Bandura, 1989). Its importance for a broad range of psychological variables is acknowledged and widely investigated (see Skinner, 1996 for a review). Regarding behavioral outcomes, perceptions of control have been included as a central construct in several theories dealing with motivation and future behavior (Control Theory: Glasser, 1984; Valence–Instrumentality–Expectancy Theory: Vroom, 1964; Theory of planned behavior: Ajzen, 1991; Theory of Learned Helplessness: Seligman, 1975; Abramson et al., 1978).

Risk-taking behavior, which can be defined as an action with uncertain consequences that might be either potentially positive or harmful (Renn, 1998), is considered to be one specific facet of decision-making behavior with an especially strong relation to perceptions of control as outlined above. Thus, it seems plausible that risk taking is influenced by prior experiences of personal control, the more so when keeping in mind the close relation of control and risk as well as the direct link between perceptions of control and risk perceptions.

However, evidence for direct effects of experiences of control on risk-taking behavior is scarce. The relationship has largely been investigated with a focus on the effects of chronic losses on future investment decisions (e.g., Rivers and Arvai, 2007). More recently initial evidence for causal effects of loss of control experiences on risk propensity was provided (Wimmer et al., 2010). In contrast to the previous studies, their experimental manipulation induced internally attributable difficulty-related loss of control (instead of assumingly chance-related chronic losses) and the subjectively experienced perceptions of loss of control were explicitly measured and thus interpretable. Results demonstrated a causal relationship between prior experiences of (internally attributed) loss of control and decreased risk-taking behavior. Apart from this initial finding, research is largely lacking. In the current study, we focus on externally attributable loss of control and additionally assume that emotions might play a significant role in this relationship.

## The Role of Emotions in the Relationship between Loss of Control and Risk Taking

It is well-known that emotions arise from personally relevant experiences of control, especially experiences with loss of control (e.g., Skinner, 1996; Goetz et al., 2010). Additionally, emotions powerfully influence decision-making processes (e.g., Loewenstein and Lerner, 2003). In particular, incidental emotions have been shown to impact decisions and thus can account for spillover effects between even objectively unrelated settings. Therefore, emotions might operate as a link between loss of control experiences and risk-taking behavior; more precisely, they may be worthwhile to examine as a potential mediator of this link.

### The Relevance of Perceptions of Control for Emotions

Cognitive emotion theories (e.g., see Lazarus et al., 1970; Scherer et al., 2001) propose that emotions are not aroused by events *per se*, but by cognitive evaluations or appraisals of the events, and the resulting discrete emotions depend on this pattern of appraisals. Several dimensions underlying these appraisals have been discussed, among which control appraisals play a prominent role. In various appraisal theories of emotions the dimension of control is consistently identified as one of the central appraisal dimensions and thus is regarded as a central antecedent of emotions (e.g., Scherer, 1982; Smith and Ellsworth, 1985; Roseman et al., 1996).

#### Appraisal Dimension of Control and the Valence of Emotions

The control-value theory (Pekrun, 2000, 2006) focusing on the antecedents and development of emotions considers control to be one of the two most important appraisal dimensions in the emergence of emotions. Control-related cognitions are believed to essentially determine the valence of emotions (i.e., positive vs. negative) and appraisals of lack of control are associated with negative emotions (Pekrun, 2000). Thus, generally, experiences of loss of control due to changes in external conditions might be associated with a variety of negative emotions, such as fear, anger, or hopelessness. The concrete emotional quality arising from those experiences is influenced by additional factors, such as evaluation of the circumstances and attributions of causes (e.g., Weiner, 1985).

#### Externally Caused Subjective Loss of Control and its Emotional Consequences

This study is particularly concerned with the effects of experiences of loss of control caused by changes in external conditions that handicap one’s task performance, but that lack a personally threatening potential. Annoying technical difficulties with computer hard- or software might be the everyday counterpart of this experience. Given ongoing technological developments and our reliance on technology, this kind of experience is assumed to be ubiquitous in most people’s daily work. Thus, our study deals with one’s subjective experiences of loss of control and consequently the lack of a possibility to fulfill one’s task and to produce the desired performance.

The primary emotion arising under these circumstances is assumed to be anger. In several studies, anger has been induced by malfunctioning computer equipment (e.g., Deffenbacher et al., 1996; Why and Johnston, 2008; Deffenbacher, 2011). In contrast to fear, anger should be elicited by this kind of subjective loss of control experience determined by external causes, but lacking a personal threat. Anger is characterized as a retrospective emotion following from negative developments and outcomes (Pekrun, 2006) which are explicitly externally, not internally, attributed. According to Weiner (1985), anger is an attribution-dependent emotion implying an appraisal that one’s personal goal attainment has been blocked (Kitayama et al., 2006). In addition to these rather cognitive facets, anger is considered an activating emotion (Shaver et al., 1987; Pekrun, 1992; Kleine et al., 2005) and relates to an approach motivation to change the current situation (Carver and Harmon-Jones, 2009), which is especially interesting in regard to the context of the current study because it points toward subsequent behavior and decision making (Lerner and Keltner, 2001).

### Emotions and Their Impact on Risk-Taking Behavior

Research has shown that risk-related decisions are not fully understandable when only purely objective (“rational”) facts, such as probability and severity of possible outcomes are considered. Instead, subjective influences, such as incidental affective states (e.g., Waters, 2006), have emerged as important predictors of these decisions. Following a considerable amount of research focusing on the effects of positive and negative mood (e.g., work by Isen and colleagues; for a review see Isen, 2000), recent research has gone beyond this valence dimension of affect and instead turned toward the differential effects of specific discrete emotions, such as fear, anger, or happiness (e.g., Lerner and Keltner, 2001).

The *Appraisal Tendency Framework* (ATF; Lerner and Keltner, 2000, 2001; see also Han et al., 2007) proposed a general theoretical model describing emotion-specific impacts on economic decision making. It allows a precise prediction of the differential impact of discrete emotions on particular decision-making processes and outcomes due to their link to emotion-specific appraisal tendencies. Based on the premise of appraisal theories that each emotion is characterized by a unique appraisal pattern on central appraisal dimensions (such as pleasantness, certainty, control; see Smith and Ellsworth, 1985), the ATF assumes that “each emotion activates a cognitive predisposition to appraise future events in line with the central-appraisal dimensions that triggered the emotion” (Lerner and Keltner, 2000, p. 477). This cognitive predisposition for future appraisals is called appraisal tendency and underlies the carry-over effects by which emotions influence subsequent judgments and decision making. To exert strong influences, the emotion’s central appraisal content must be thematically linked to the decision-making topic (Lerner and Keltner, 2001).

In the context of risk-related decision making, the appraisal dimensions certainty and control are thought to be particularly influential due to their close association with cognitive evaluations for determining risk assessments. The emotion anger in particular is characterized by high appraisals of both certainty and control (Lerner and Keltner, 2000), and is therefore proposed to influence risk-related decisions. Thus, for our study’s objective of investigating the consequences of subjective loss of control experiences on risk-taking behavior, the effects of anger are particularly interesting and thus central to our focus – and might appear counterintuitive at a first glance: Despite originally being elicited by loss of control experiences (which in this study’s experimental manipulation are due to externally attributable changes of objectively given circumstances), the emotional experience of anger is subsequently assumed to be accompanied by high certainty and control appraisals. These accompanying appraisal tendencies are then expected to impact subsequent risky decision making. Drawing from both the ATF (due to high levels of certainty and control) and the notion of anger being an activating emotion characterized by features associated with approach motivation (Lerner and Tiedens, 2006; Carver and Harmon-Jones, 2009), anger is believed to increase people’s risk-taking decisions. Therefore, because our experimental paradigm was designed to elicit externally attributed subjective loss of control and thus, anger, we assume anger will mediate the impact of loss of control experiences on risk-taking behavior.

## Cultural Influences and Cross-Cultural Universality

When considering previous studies on the variables we are interested in, namely control experiences, anger, risk taking, and their underlying mechanisms, such as causal attributions and cognitive appraisals, cultural differences seem to be a relevant issue. However, so far, the research on the cultural influences of some of these aspects provides rather mixed results. Whereas evidence on differences in behaviors, perceptions of, and attitudes toward risks is not unambiguous (Bontempo et al., 1997; Weber and Hsee, 1998; Rohrmann and Chen, 1999; Brumagim and Wu, 2005), cross-cultural variations seem to be particularly pertinent with respect to emotions and control perceptions, especially when comparing individualistic and collectivistic cultures (for definition of cultural value dimensions, see Triandis, 1994, 1995; Hofstede, 2001).

With respect to control-related constructs, culture seems to influence levels and patterns of general control beliefs (e.g., internal vs. external locus of control, primary vs. secondary control). For example, people from Western European countries seem to habitually perceive higher levels of personal, thus internal, control than people from Eastern Asian countries (e.g., see Spector et al., 2004). In addition to the individual level, there might also be differences with respect to the extent of perceived power, control, and uncertainty at the country level (cf., cultural dimensions by Hofstede, 2001, such as power distance index, uncertainty avoidance index). However, the personal need for being able to control one’s environments is regarded as a fundamental motive (Heckhausen and Schulz, 1995; Skinner, 1996). Correspondingly, the consequences of experiencing gains in, maintenance of, or loss of control over personally relevant situations or outcomes is considered universal and generalizable across cultures.

Similarly, various facets of emotions have been shown to be culturally influenced, for example, emotional expressivity, norms for feeling and displaying emotions, and interpretations and interpersonal consequences of emotions (e.g., Markus and Kitayama, 1991; Eid and Diener, 2001; Mesquita and Walker, 2003; Van Hemert et al., 2007; Matsumoto et al., 2008). These facets are – at least partly – able to explain cultural differences in the frequency and intensity of emotions.

However, without questioning potential mean level differences between cultures in various variables, basic relations between constructs are typically considered to be cross-culturally valid (also see Frenzel et al., 2007a,b for universality of constructional associations despite mean level differences). For example, “cognitive-affective linkages” between attributions and resulting emotions are not found to differ cross-culturally (Brown and Cai, 2010, p. 111). Pekrun (2006, p. 329) also lends support for relative cross-cultural universality stating “general functional mechanisms of human emotions are bound to universal, species-specific characteristics of our mind.” Further, previously mainly collective cultures are becoming more and more individualistic, especially in those parts of the world where the economy is growing (e.g., China), which continually reduces differences between collectivistic and individualistic countries (Hamamura, 2012; Steele and Lynch, 2013).

Instead of focusing on absolute levels or the intensity of control and emotional experiences, in this study the effects of loss of control experiences on risk-taking behavior and the role of anger in this relationship are at issue. More precisely, we will explore whether these relational and functional associations are cross-culturally generalizable. For this purpose, the effects of subjective loss of control experiences on anger and risk-taking behavior will be investigated using samples from two countries, namely Germany and China, which represent differing cultural backgrounds and which are characterized by numerous culturally determined differences, also with respect to mean levels in the variables of interest.

## Research Aims and Hypotheses

In summary, the objectives of this set of studies are as follows: first, the impact of subjective experiences of loss of control due to external changes in control conditions on subsequent risk-taking behavior will be examined. Second, the role of anger in this relationship will be investigated. Third, the cross-cultural generalizability of the proposed relationships between subjective loss of control, anger, and risk-taking behavior will be explored.

The first aim is to develop an appropriate experimental paradigm for inducing externally attributable subjective loss of control (see Pilot Experimental Study) in order to allow for the investigation of the proposed hypotheses. Following the development of the experimental paradigm, the hypotheses concerning the effects of loss of control on anger and risk-taking behavior are investigated (cf., Main Experimental Study 1) and the cross-cultural generalizability of the proposed relationships are explored with participants from different cultural (individualistic vs. collectivistic) backgrounds (cf., Main Experimental Study 2):

Hypothesis 1: Externally caused and consequently externally attributed loss of control experiences are assumed to cause anger.
Hypothesis 2: Subjective loss of control experiences which are attributed to external causes are assumed to increase subsequent risk-taking behavior.
Hypothesis 3: The relationship between externally caused and consequently externally attributed loss of control experiences and increased risk-taking behavior is assumed to be mediated by anger.
Hypothesis 4: The mediating effect of anger on the relationship between subjective loss of control and risk-taking behavior is assumed to be cross-culturally generalizable.

## Pilot Experimental Study

### Aims

The objective of this Pilot Experimental Study was to test the effects of our newly developed experimental manipulation of objective control conditions which was designed to induce a sense of subjective loss of control due to external causes. This pilot study aimed to investigate the assumed effects of a manipulation check in order to ensure the paradigm’s adequacy to explore the previously proposed hypotheses in the subsequent experiments.

### Method

#### Participants and Data Collection

##### Sample

*N* = 44 German university students (50% female) with an average age of *M* = 21.52 years (*SD* = 1.98, range: 19–27) voluntarily participated in this study. The participants were recruited using the online recruiting system ORSEE (Greiner, 2004) and they were compensated by a fixed show-up (9 asar) fee plus payment according to their individual performance in the problem-solving game (theoretical range: 0–8 asar). The assignment to the treatment conditions was random with *n* = 22 (50% female) participants in the experimental (EG) and *n* = 22 (50% female) participants in the control group (CG).

##### Procedure and experimental design

**FIGURE 1:**
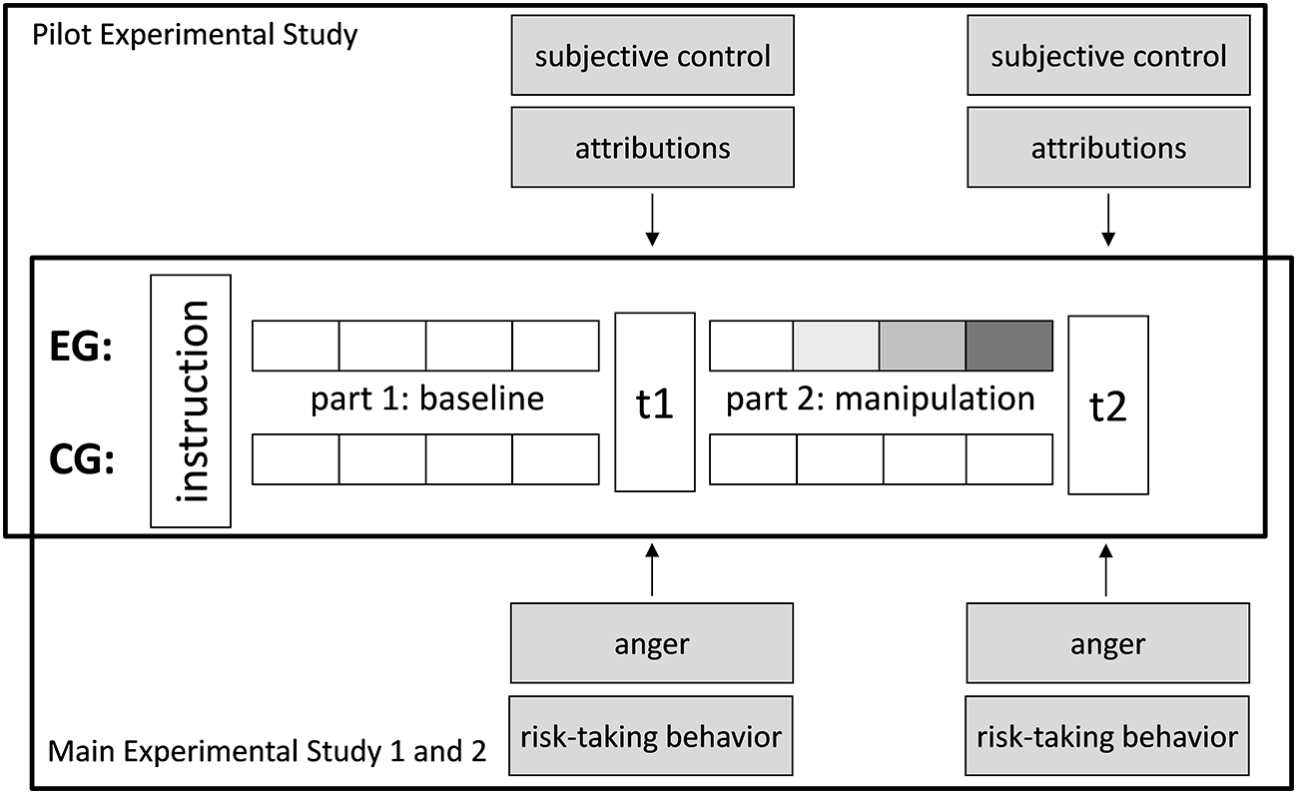
**One-factor pre–post design of Pilot Experimental Study and Main Experimental Studies 1 and 2**. The Pilot Experimental Study tested the experimental paradigm’s adequacy for inducing subjective loss of control. By decreasing the accuracy with which the participants’ prediction was implemented into the computer game, the experimental group’s (EG’s) objectively given control was increasingly reduced during the four rounds of the manipulation section (represented by the darkening grey color). In a one-factor pre–post design the EG and control (CG) group’s subjective control ratings and external attributions following the baseline (t1) and manipulation (t2) sections of the experiment were compared. Main Experimental Studies 1 and 2 applied the same experimental paradigm to investigate the effects of loss of control on anger and risk-taking behavior in Germany and China, respectively.

**FIGURE 2:**
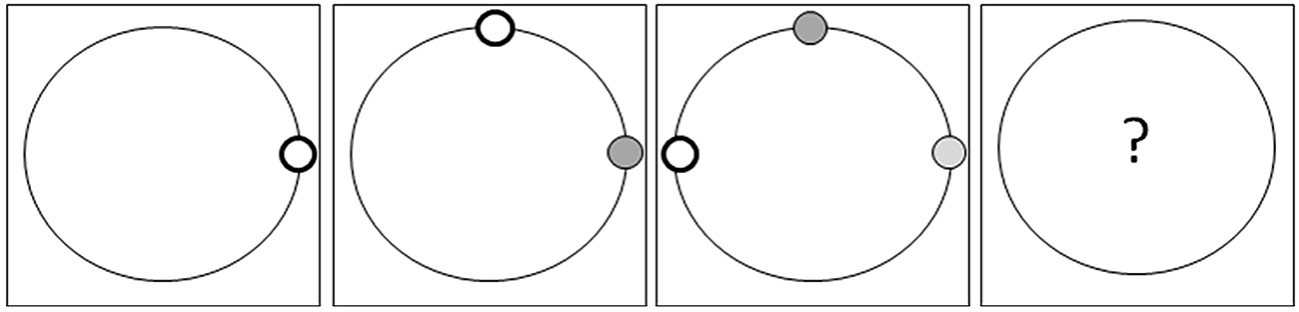
**A sample schematic representation of the computer-based problem-solving task**. Participants are asked to continuously indicate the assumed next position of the little white circle while its previous positions fade from dark gray to lighter shades of gray.

The experimental study consisted of a one-factor pre–post design with questionnaires following the baseline and manipulation sections (cf., **Figure 1**). Following an instruction phase that included a comprehension test, both the EG and CG played eight rounds of an incentivized computer-based problem-solving task (maximum profit: 1 asar per round) in which the participants had to predict by mouse click where an object would be displayed on a circle by recognizing the systematic pattern underlying the previously displayed objects (for an example, see **Figure 2**). The participants were paid according to their correct prediction of the objects’ positions (prediction accuracy was transferred into a monetary reward with the maximum precision being compensated by 1 asar per round). The patterns were determined based on the angular distances of the subsequently displayed objects; this is similar to a continuing number series or patterns task which is frequently used to test non-verbal reasoning in common tests of intelligence (for example, see the K-ABC-II, Kaufman and Kaufman, 2004; CogAT6, Lohman and Hagen, 2001; CFT-20-R, Weiβ, 2006).

**FIGURE 3:**
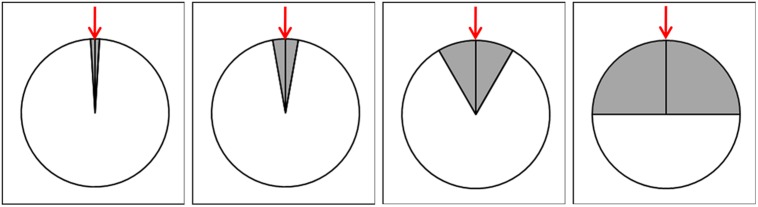
**A sample schematic representation of the experimental decreasing accuracy manipulation of displaying the participants’ prediction of the next position**. The range of the intervals in which the clicking position was displayed was ±3.5° centered around the actual chosen position (red arrow) in round 5, ±10° in round 6, ±30° in round 7, and ±90° in round 8.

In order to avoid deception the written instruction before the start of the experiment informed participants that unexpected events may occur. The first four rounds represented the baseline section in which both the CG and EG were supposed to experience subjective control; this section did not differ between the groups. The second four rounds belonged to the manipulation section and the objectively given control was continuously reduced for the participants in the EG. This induced loss of control was obtained by decreasing the accuracy with which the participants’ prediction of the object’s next position was implemented into the computer game. Instead of displaying the participant’s clicking position accurately, it was displayed randomly within an interval including the chosen position. The range of the interval increased gradually from ±3.5° in round 5, to ±10° (round 6), then ±30° (round 7), and finally to ±90° in round 8 (cf., **Figure 3**). This computer-based paradigm was designed using the Zurich Toolbox for Readymade Economic Experiments (*z*-Tree; Fischbacher, 2007) as experimental software.

Between the baseline and manipulation section (t1) as well as at the end of the manipulation section (t2) the participants answered a questionnaire on their subjective perceptions of control and on their attributions for the perceived control. Furthermore, in order to ensure general comparability between the EG and CG, during a separate follow-up attended by each participant within about 2 weeks following the experimental part of the study, socio-demographic (sex, age, subject of study, mother language, pre-experiences with computer-games) and several potentially relevant trait variables (non-verbal reasoning: German intelligence test I-S-T 2000-R by Liepmann et al., 2007, English version: Beauducel et al., 2010; locus of control: IPC by Levenson, 1972, German version by Krampen, 1981) were assessed^1^.

#### Variables and Study Measures

##### Subjective control

The participants’ subjective perceptions of control over their outcomes with respect to the previously played round were assessed twice, following both the baseline (t1) and manipulation section (t2). The items, based on the Academic Control Scale (Perry et al., 2001), were adapted to the experimental context (e.g., “I could completely determine my outcomes”) and rated on a seven-point-rating-scale ranging from 0 *completely disagree* to 6 *completely agree*. Cronbach’s alphas of the two-item-measure were α = 0.58 (t1) and α = 0.88 (t2).

##### Attributions

In order to control whether the experimentally manipulated changes in control were in fact attributed to external causes by the participants as intended, their attributions were assessed following the baseline and manipulation section. The participants indicated to what extent they attributed their perceived control to either internal or external causes (e.g., external attribution: “My outcomes depended on influences lying outside myself”) on seven-point-rating-scales ranging from 0 *completely disagree* to 6 *completely agree*. The internal consistencies were α = 0.72 (t1) and α = 0.96 (t2) versus α = 0.68 (t1) and α = 0.87 (t2) for the two-item external versus internal attribution scales, respectively.

### Results

#### Subjective Control

**FIGURE 4:**
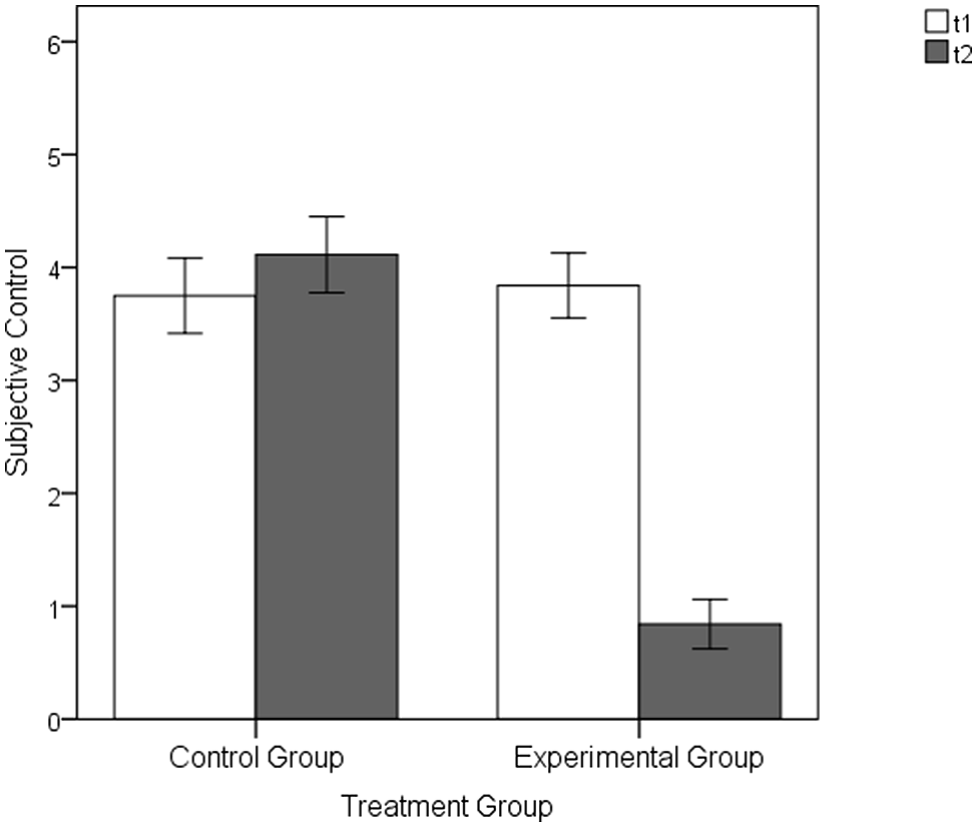
**Subjective control ratings of the experimental and CG following the baseline (t1) and the manipulation (t2) part of the Pilot Experimental Study**. Error bars represent SEM (±1 *SE*).

Following the baseline part at t1 there were no significant group differences in the subjective control evaluations between the treatment conditions, EG: *M* = 3.84, *SD* = 1.35 versus CG: *M* = 3.75, *SD* = 1.56; *t*(42) = 0.21, *p* = 0.837, *d* = 0.06. However, in line with our expectations, the EG rated their subjective control significantly lower than the CG following the experimental manipulation at t2, *t*(42) = -8.14, *p* < 0.001, *d* = -2.46 (EG: *M* = 0.84, *SD* = 1.03; CG: *M* = 4.11, *SD* = 1.58), see **Figure 4**.

#### Attributions

**FIGURE 5:**
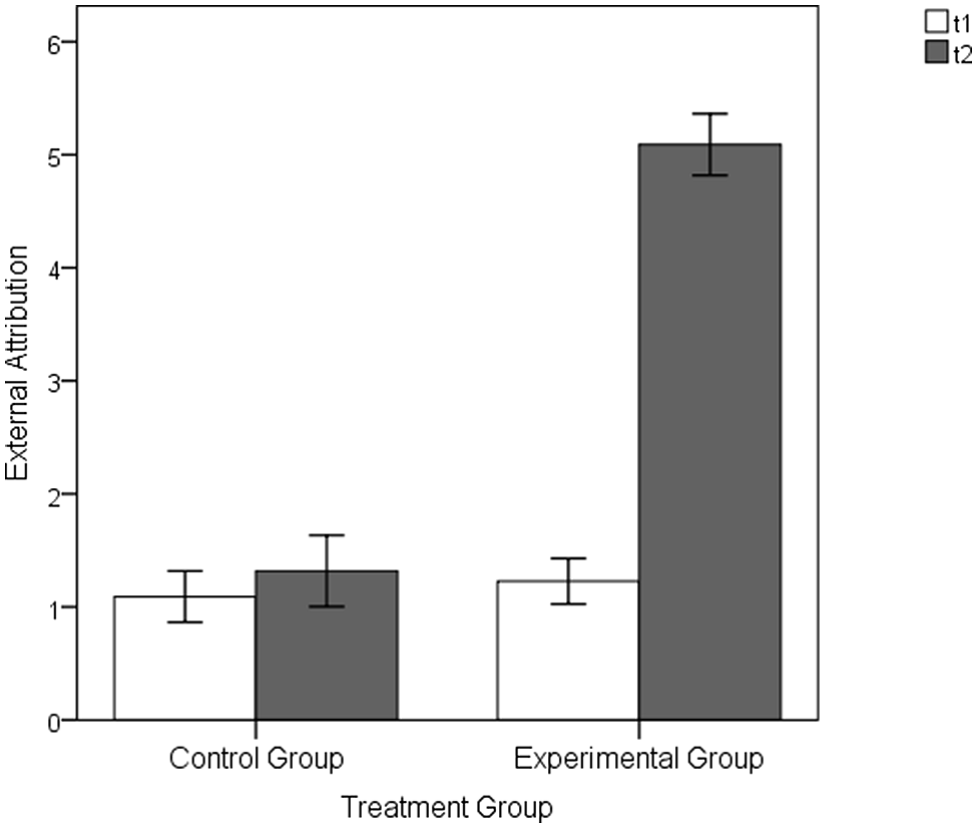
**External attributions of the EG and CG following the baseline (t1) and the manipulation (t2) part of the Pilot Experimental Study**. Error bars represent SEM (±1 *SE*).

**FIGURE 6:**
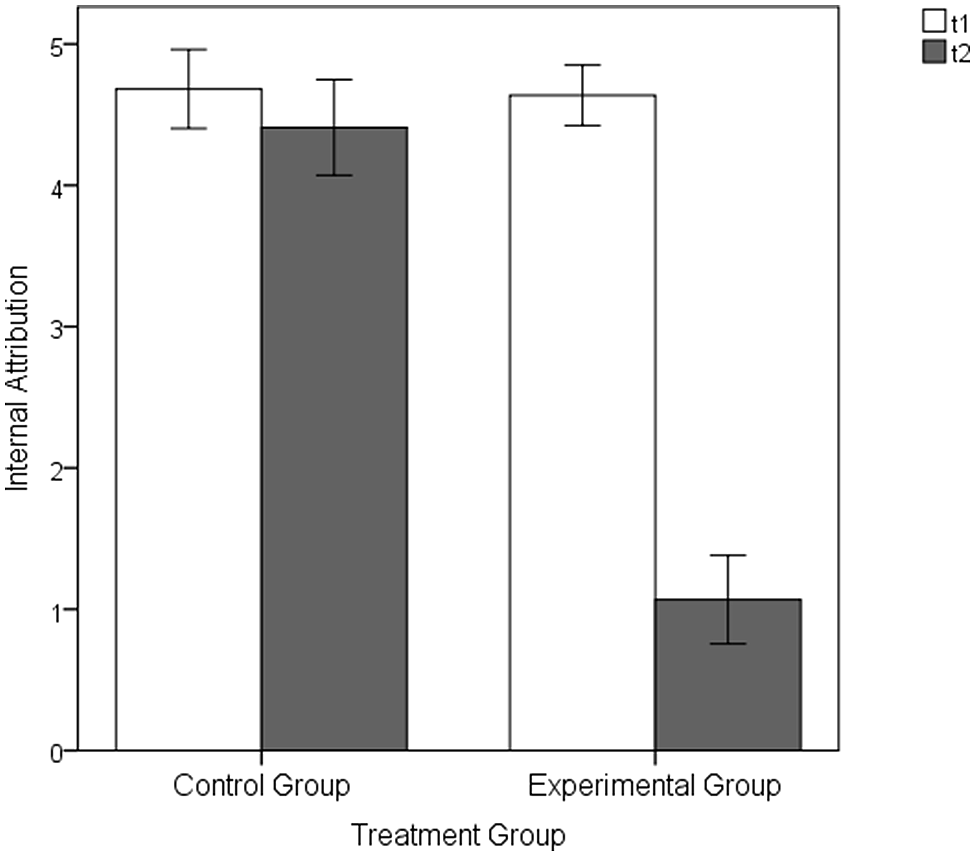
**Internal attributions of the EG and CG following the baseline (t1) and the manipulation (t2) part of the Pilot Experimental Study**. Error bars represent SEM (±1 *SE*).

The analyses of the attribution ratings showed that before the experimental manipulation at t1 there was no significant mean group differences, neither in the extent of external attributions, EG *M* = 1.23, *SD* = 0.95 versus CG: *M* = 1.09, *SD* = 1.06; *t*(42) = 0.45, *p* = 0.656, *d* = 0.14, nor in the extent of internal attributions, EG *M* = 4.64, *SD* = 1.00 versus CG: *M* = 4.68, *SD* = 1.31; *t*(42) = -0.13, *p* = 0.898, *d* = -0.04. However, following the experimental manipulation at t2, the participants in the EG attributed the perceived control to be significantly more external than the participants in the CG [external attribution scale: EG: *M* = 5.09, *SD* = 1.28 versus CG: *M* = 1.32, *SD* = 1.48; *t*(42) = 9.06, *p* < 0.001, *d* = 2.73; see **Figure 5**; internal attribution scale: EG: *M* = 1.07, *SD* = 1.47 versus CG: *M* = 4.41, *SD* = 1.59; *t*(42) = -7.26, *p* < 0.001, *d* = -2.19; see **Figure 6**]. While the extent of internal and external attributions stayed constant between t1 and t2 in the CG (internal attribution: *p* = 0.389, *d* = 0.19; external attribution: *p* = 0.448; *d* = -0.17), the experimental manipulation caused highly significant changes in attributions within the EG: The internal attributions of control decreased (*p* < 0.001, *d* = 2.34), whereas the external attributions increased (*p* < 0.001, *d* = -2.79).

### Discussion

In this study the expected impact of the experimental manipulation of objectively given control on attributions and the subjectively perceived extent of control was supported. For the participants of the EG, the manipulation applying decreasingly accurate displays of the participant’s clicking position within the computer-game led to a sense of subjective loss of control compared to the baseline section and the CG. Furthermore, the external attributions of perceived control significantly increased following the experimental manipulation whereas the internal attributions significantly decreased. The attributional pattern of the EG shows the intended dominance of external compared to internal attributions following the experimental manipulation at t2. Thus, this study’s results provide evidence for the expected impact of decreasing objective control on subjective control ratings and provide support for the paradigms’ adequacy to induce subjective loss of control experiences due to external causes.

## Main Experimental Study 1

### Aims

Main Experimental Study 1 aimed at investigating the impact of externally caused subjective loss of control on anger and risk-taking behavior (Hypotheses 1–3) by applying the newly developed and tested experimental paradigm inducing subjective loss of control by decreasing the implementation accuracy during the computer-based problem-solving task.

### Method

#### Participants and Data Collection

##### Sample

The study was conducted at a German university using a student sample consisting of *N* = 84 (54% female) participants recruited via the online recruiting system ORSEE (Greiner, 2004). The participants had an average age of *M* = 21.93 years (*SD* = 1.93, range: 19–27) and they were randomly assigned to the EG (*n* = 44; 57% female) and the CG (*n* = 40; 50% female). Their voluntary participation was compensated by a fixed show-up fee (11 asar) as well as additional payment according to the subject’s performance during the problem-solving task and risk game (theoretical range: 0–20.40 asar).

##### Procedure and experimental design

Main Experimental Study 1 used the experimental computer-based game paradigm pretested in the Pilot Experimental Study. However, instead of measuring the participants’ perceived control and attributions, following the baseline (t1) and manipulation section (t2) their anger was assessed by a questionnaire. Additionally, the participants’ behavior in a risky situation was investigated (cf., **Figure 1**). Finally, a separate follow-up during an about 2-week-interval after the experimental part of the study was used to assess the socio-demographic and trait variables (non-verbal reasoning, locus of control, as well as trait-based risk-taking propensity: DOSPERT by Weber et al., 2002, German version by Johnson et al., 2004)^2^.

#### Variables and Study Measures

##### Anger

Applying the subscale of the Differential Emotions Scale (DES; Izard et al., 1974; as cited in Izard, 1977; German version: Merten and Krause, 1993) consisting of three adjective items (“enraged,” ”angry,” “mad”), participants’ anger was assessed by their ratings on a five point intensity rating scale ranging from 0 *not at all* to 4 *very strong* (e.g., “To what extent do you experience these feelings at the moment?” “I feel…” “angry,”…)^3^. The three adjective-subscale had an internal consistency of α = 0.84 at t1 and α = 0.94 at t2.

##### Risk-taking behavior

The participants’ risk-taking behavior was assessed by computer-based variants of the “devil’s task” (Slovic, 1966) consisting of a circle with a given number of equal sectors. All – except one or two – of those sectors represent “secure” sectors, whereas the remaining one or two sectors are the “devil’s” sectors. The subjects knew how many sectors were secure and “devil’s” sectors, but their positions were unknown to the participants. While choosing a secure sector resulted in a gain of 0.10 asar per sector, choosing a devil’s sector caused the loss of all money in this round. The participants were allowed to decide on both the number and position of fields they could choose. This task reflects a typical risk situation with the number of chosen fields serving as the (continuous) dependent variable with a theoretical range between 0 and 23 or 31 sectors depending on the version of the game: The participants were presented one version of this game at t1 (31 sectors, one devil’s sector), and three immediately succeeding versions at t2 (game 1: 23 sectors with one devil’s sector; game 2: 31 sectors, two devil’s sectors; game 3: 23 sectors, two devil’s sectors). The reason for using different variants of the devil’s task was to avoid memory effects and thus making the measurement more reliable. In order to avoid any effects on the subsequent versions of the devil’s task, the participants did not receive any immediate feedback on their results between the rounds, but only at the end of the experiment. The internal consistency of the three versions of the devil’s task at t2 was α = 0.86.

### Results

#### Anger

**FIGURE 7:**
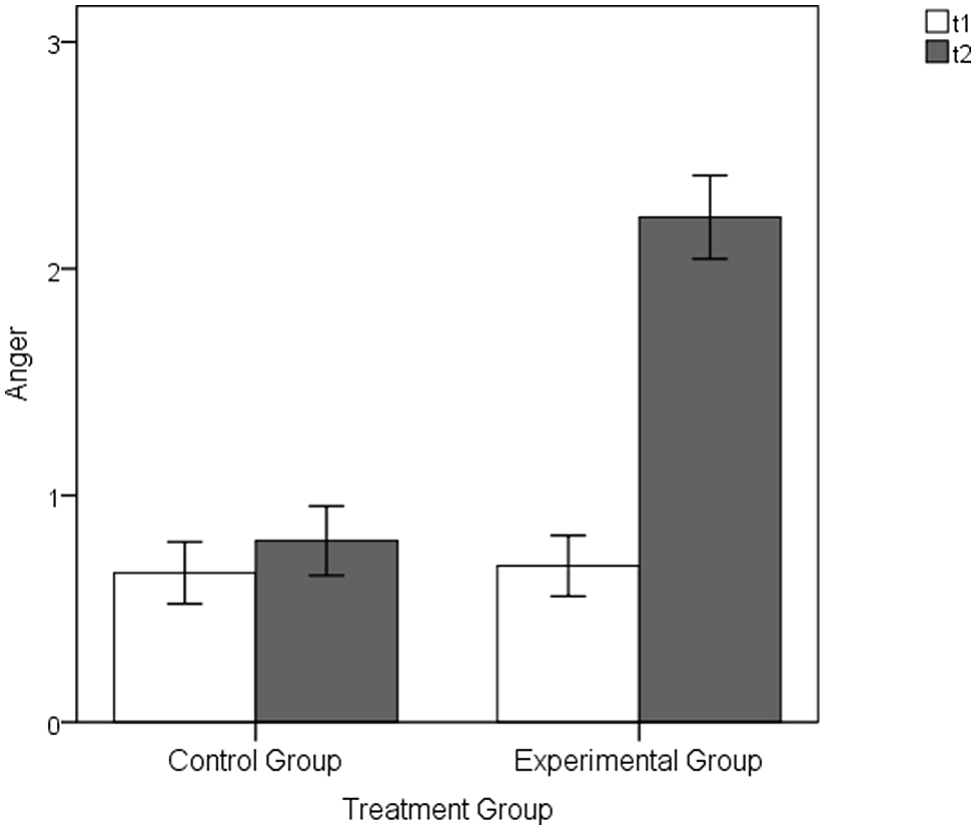
**Anger ratings of the EG and CG following the baseline (t1) and the manipulation (t2) part of the Main Experimental Study 1**. Error bars represent SEM (±1 *SE*).

In line with our assumptions there were no significant group differences before the manipulation at t1 [CG: *M* = 0.66, *SD* = 0.87, EG: *M* = 0.69, *SD* = 0.89, *t*(82) = -0.16, *p* = 0.872, *d* = -0.04]. In contrast, and supporting Hypothesis 1, the members of the EG showed significantly higher levels of anger following the externally attributable subjective loss of control manipulation (*M* = 2.23, *SD* = 1.22) than the participants in the CG (*M* = 0.80, *SD* = 0.97), *t*(82) = -5.90, *p* < 0.001, *d* = -1.29 (see **Figure 7**).

#### Risk-Taking Behavior

**FIGURE 8:**
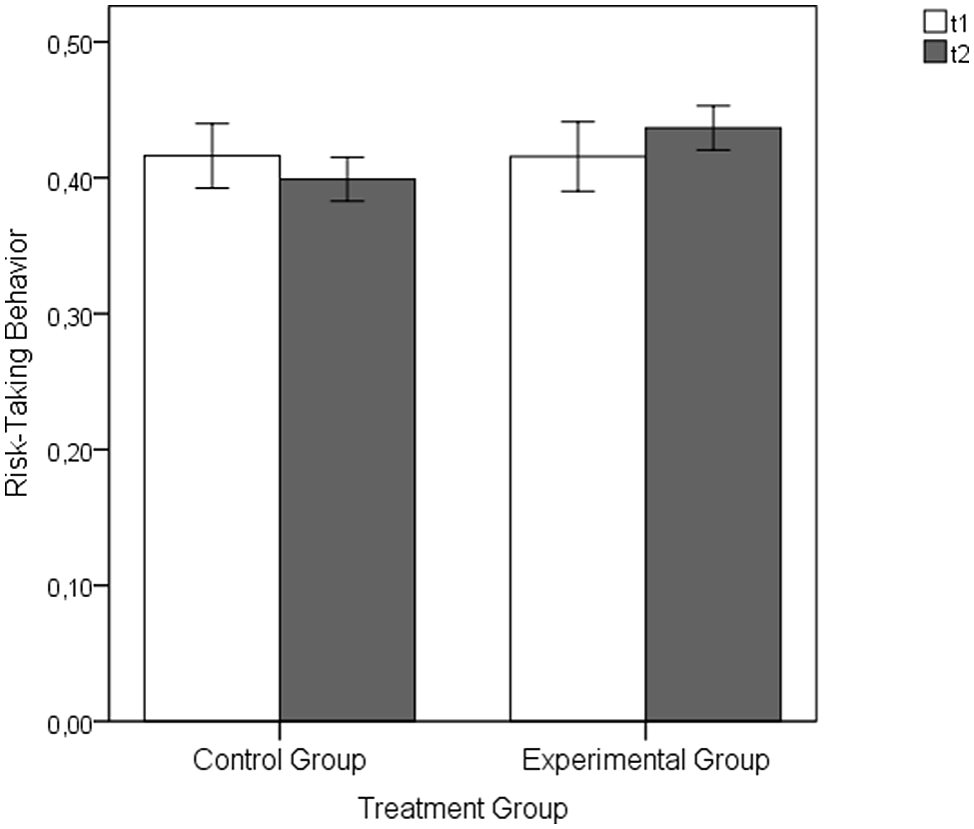
**Risk-taking behavior of the EG and CG represented by the mean proportion of chosen sectors during the devil’s task following the baseline (t1) and the manipulation (t2) part of the Main Experimental Study 1**. Error bars represent SEM (±1 *SE*).

Similarly, there was no group difference with respect to the risk-taking behavior in the baseline section (average proportion of chosen sections in the devil’s task; theoretical range: 0–1) at t1: CG: *M* = 0.42, *SD* = 0.15, EG: *M* = 0.42, *SD* = 0.17, *t*(82) = 0.01, *p* = 0.990, *d* = 0.002. Following the experimental manipulation, the EG tended to take more risks as compared to the CG (see **Figure 8**). During the three rounds of the devil’s task the EG’s proportion of chosen sections on average was *M* = 0.44 (*SD* = 0.11), while the CG’s was *M* = 0.40 (*SD* = 0.10). This group difference was marginally significant, *t*(82) = -1.64, *p* = 0.053, with *d* = -0.36 representing a small effect size (Cohen, 1988) and providing partial support for Hypothesis 2.

#### Anger as a Mediator of the Relationship between Subjective Loss of Control and Risk-Taking Behavior

**FIGURE 9:**
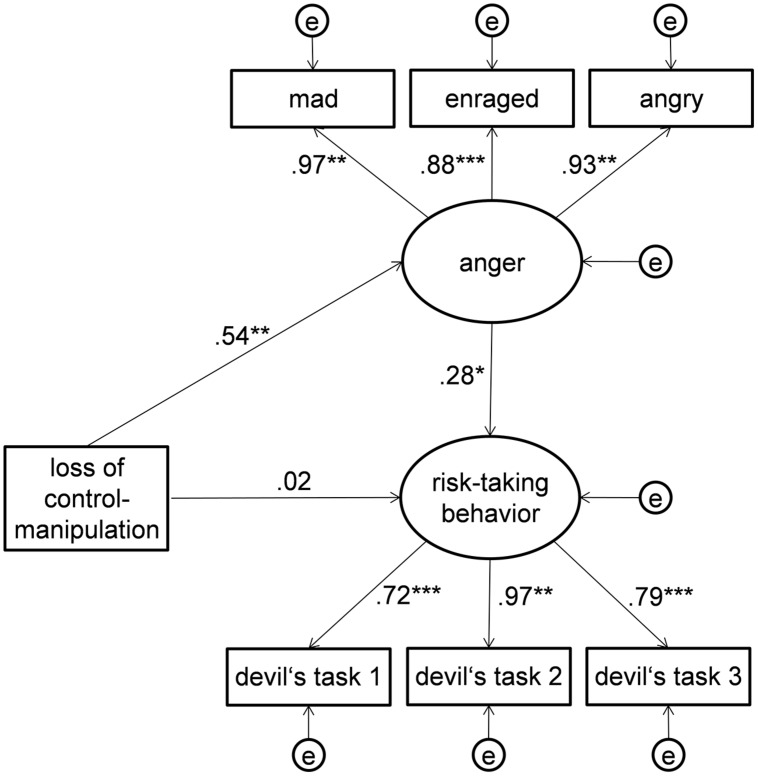
**Structural equation model for testing the indirect effect (Hypothesis 3) of Main Experimental Study 1 on a latent variable level**. The coefficients represent standardized factor loadings and standardized regression paths in the German sample (*N* = 84). ^∗^*p* < 0.05, ^∗∗^*p* < 0.01, ^∗∗∗^*p* < 0.001.

In order to examine the mediating effect of anger on the relationship between the subjective loss of control manipulation and subsequent risk-taking behavior as postulated in Hypothesis 3, we applied structural equation modeling (SEM) techniques (see Byrne, 2010; Kline, 2010) which provide excellent methods for testing indirect effects. The proposed mediation was modeled with anger and risk-taking behavior as latent variables (cf., **Figure 9**). The three items of the anger subscale of the DES were modeled as manifest indicators of the latent variable anger. Similarly, the mean proportion of chosen sectors in the three devil’s task rounds following the experimental manipulation section at t2 served as manifest indicators of the latent variable risk-taking behavior. The proposed mediating effect was modeled by the three unidirectional paths leading from the manifest variable, subjective loss of control manipulation (independent variable), toward the latent variables anger and risk-taking behavior as well as from the latent variable anger (mediator) toward the latent variable risk-taking behavior (dependent variable).

The results of the CFA showed that the proposed model was not rejected in the test of significance (χ^2^= 17.21, *df* = 12, *p* = 0.142) and showed very satisfactory model fit indexes (CFI = 0.987; RMSEA = 0.072; SRMR = 0.046) which are in line with the recommended cutoff criteria for acceptable model fit (Hu and Bentler, 1999). The standardized indirect effect of the subjective loss of control manipulation on risk-taking behavior via anger in this model was 0.15, *p* = 0.013. The Sobel test for the latent regression path coefficients was significant with *z* = 2.03, *p* = 0.042.

Thus, the analyses of indirect effects supported the mediating effect of anger proposed in Hypothesis 3. Furthermore, the confirmatory factor analysis added evidence for a very satisfactory general fit of the proposed model.

### Discussion

This study provided evidence for the assumed effects of externally caused subjective loss of control on anger and risk-taking behavior. As expected, following the externally attributable experimental manipulation of objective control, the participants reported increased levels of anger and tended to act more riskily in a subsequent decision-making setting. The group difference with respect to the risk-taking behavior was only marginally significant (e.g., Cohen, 1992). Further, the effect size of the mean difference which – according to Cohen’s (1988) guidelines – is regarded as a small effect. In sum, the empirical data supported the assumed mediating role of anger on the relationship between subjective loss of control and risk-taking behavior. In conclusion, the results of Main Experimental Study 1 were in line with our expectations and provided supporting evidence for Hypotheses 1–3.

## Main Experimental Study 2

### Aims

The objective of Main Experimental Study 2 was to test Hypothesis 4 which proposes the assumed mediating mechanism of anger on the relationship between subjective loss of control and risk-taking behavior will be generalizable cross-culturally. For this reason the Main Experimental Study 1 was replicated within a Chinese sample^4^ to allow for cross-cultural comparisons.

### Method

#### Participants and Data Collection

##### Sample

*N* = 125 (64% female) Chinese students participated in the replication study. The average age was *M* = 20.44 years (*SD* = 1.78, range: 17–28). The participants were randomly assigned to the EG (*n* = 61; 61% female) and the CG (*n* = 64; 67% female) and compensated by a fixed show-up fee (48 RMB) with additional payment given according to their performance in the problem-solving and risk game (theoretical range: 0–91.80 RMB).

##### Procedure, variables, and experimental design

Main Experimental Study 2 applied the same procedures, measures, and experimental design in the Chinese sample as the Main Experimental Study 1 in the German sample (cf., **Figure 1**). Thus, following the baseline (t1) and the manipulation part (t2) of the experimental design, anger as well as risk-taking behavior were assessed. To create a Chinese version of the anger subscale of the DES (Izard et al., 1974; as cited in Izard, 1977), the German version (Merten and Krause, 1993) was subjected to a multiple stage translation process consisting of independent forth- and back-translations by two professional translators, as well as comparisons, revisions, and a pretest with Chinese students. The internal consistency of the translated three-items-anger subscale was α = 0.84 at t1 and α = 0.90 at t2. Identical to the Main Experimental Study 1, the participants’ risk-taking behavior was assessed by one or three rounds of the devil’s task (Slovic, 1966) at t1 and t2, respectively. The internal consistency of the three versions of the devil’s task at t2 was α = 0.91. In accordance with the Chinese currency, choosing one secure sector resulted in a gain of 0.45 RMB and the maximum profit in the computer-based problem-solving game was 4.50 RMB per round. Socio-demographic and trait variables were assessed later the same day during a separate part following the experimental section of the study in order to allow for trait-based comparability between the EG and CG.

### Results

#### Anger

In line with our assumptions there were no significant group differences before the manipulation at t1 [CG: *M* = 0.09, *SD* = 0.26, EG: *M* = 0.16, *SD* = 0.44, *t*(123) = -1.09, *p* = 0.280, *d* = -0.07]. In contrast, and supporting Hypothesis 1, the members of the EG showed significantly higher levels of anger following the externally attributable subjective loss of control manipulation (*M* = 1.22, *SD* = 0.97) than the participants in the CG (*M* = 0.32, *SD* = 0.55), *t*(123) = -4.99, *p* < 0.001, *d* = -0.70.

Considering the cultural differences of intensity of anger between the two samples in China and Germany, the Chinese anger ratings were lower than the German ratings at both t1 [China: *M* = 0.12, *SD* = 0.36, Germany: *M* = 0.67, *SD* = 0.87, *t*(207) = -6.32, *p* < 0.001, *d* = -0.55] and t2 [China: *M* = 0.66, *SD* = 0.85, Germany: *M* = 1.55, *SD* = 1.31, *t*(207) = -5.89, *p* < 0.001, *d* = -0.88].

#### Risk-Taking Behavior

Similarly, there was no group difference with respect to the risk-taking behavior in the baseline section (average proportion of chosen sections in the devil’s task; theoretical range: 0–1) at t1: CG: *M* = 0.40, *SD* = 0.21, EG: *M* = 0.41, *SD* = 0.21, *t*(123) = 0.01, *p* = 0.397, *d* = -0.010. Following the experimental manipulation, during the three rounds of the devil’s task the EG’s proportion of chosen sections on average was *M* = 0.38 (*SD* = 0.15), and the CG’s was *M* = 0.35 (*SD* = 0.19). This group difference was in the assumed direction but did not reach significance, *t*(123) = -1.20, *p* = 0.116, with *d* = -0.037.

#### Anger as a Mediator of the Relationship between Subjective Loss of Control and Risk-Taking Behavior

Applying the multiple group comparison procedure we specified a sequence of three models with nested structures and increasing constraints of equality in order to test the invariance of the proposed models between the two samples in Germany and China. Model 1 (baseline model, identical to the model testing the mediation effect on a latent variable level in Main Experiment 1) tested for the invariance of the model form without any constraints. Model 2 included constrained factor loadings assuming that the measurement weights of the latent variables anger and risk-taking behavior on their manifest indicators operate equivalently across the two samples. Model 3 additionally tested for the invariance of the regression paths between the independent variable, mediating variable and dependent variable proposing the relationships to be comparable in both samples. In order to evaluate the adequacy of the models and their included invariance assumptions, the absolute as well as relative model fit (changes in model fit due to additionally imposed constraints) were considered (e.g., Little, 1997; Byrne, 2010). Goodness-of-fit statistics and indexes of the three models are displayed in **Table 1**.

**Table 1:** Model fit statistics and indexes of the three nested models of multiple group analysis with the German and Chinese sample.

	Model fit				
	χ^2^	*df*	*P*	CFI	RMSEA
Model 1 Invariance of model form	30.70	24	0.163	0.993	0.037
Model 2 Invariance of factor loadings	39.82	28	0.069	0.988	0.045
Model 3 Invariance of regression paths	46.14	31	0.039	0.984	0.049

*N(Germany) = 84, N(China) = 125. All p-values refer to one-tailed tests*.

**FIGURE 10:**
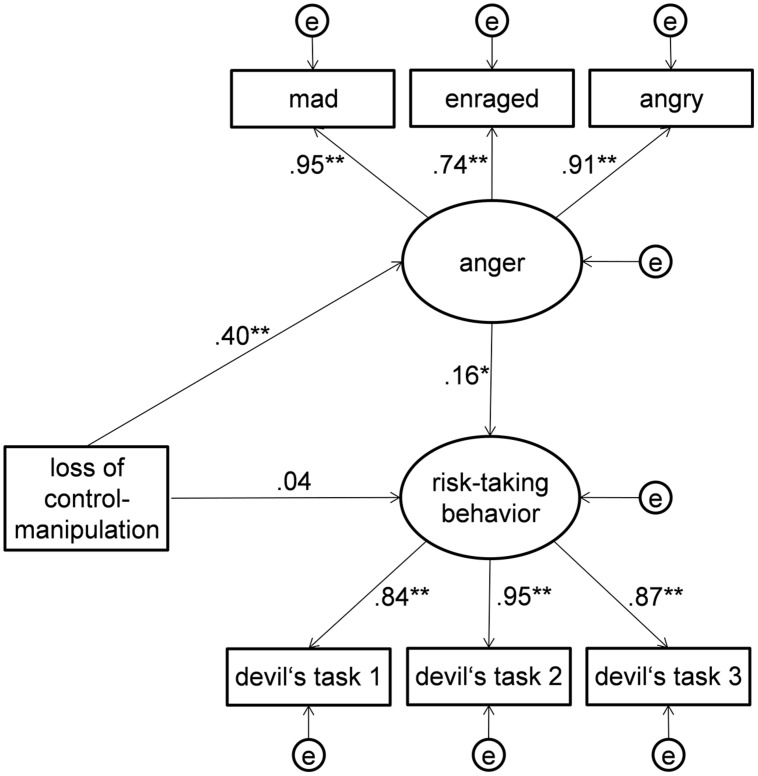
**Structural equation model for testing the indirect effect on a latent variable level in Main Experimental Study 2**. This model also served as baseline model (Model 1) for testing the cross-cultural generalizability (Hypothesis 4). The coefficients represent standardized factor loadings and regression paths of the baseline model 1 in the Chinese sample (*N* = 125). ^∗^*p* < 0.05, ^∗∗^*p* < 0.01.

Additionally, within the proposed models the indirect effects of the subjective loss of control manipulation on risk-taking behavior via anger were analyzed and tested for significance based on bootstrapping procedures. The results of the indirect effect analyses for all models and both samples are presented in **Table 2**. The results revealed a good overall fit for the baseline Model 1 [χ^2^(24) = 30.70, *p* = 0.163; CFI = 0.993; RMSEA = 0.037] and the indirect effects reached significance both in the German (standardized indirect effect: 0.15, *p* = 0.009, one-tailed, due to the directed effect) and Chinese sample (standardized indirect effect: 0.06, *p* = 0.039, one-tailed, due to the directed effect). Thus, the assumed mediating effect was supported and the invariance of the model form was confirmed in both samples. The Chinese sample’s standardized coefficients of the model are presented in **Figure 10**.

**Table 2:** Tests of standardized indirect effects within the three nested models of multiple group analysis in the German and Chinese sample.

	Germany		China	
	Indirect effect	*p*	Indirect effect	*p*
Model 1 Invariance of model form	0.15	0.009	0.06	0.039
Model 2 Invariance of factor loadings	0.15	0.009	0.06	0.036
Model 3 Invariance of regression paths	0.10	0.003	0.06	0.003

*N(Germany) = 84, N(China) = 125. All p-values refer to one-tailed tests. The tests of significance of the standardized indirect effects (ab paths) were performed based on 5000 bootstrap samples and bias-corrected 95% confidence intervals*.

Constraining the factor loadings to be invariant in Germany and China caused a marginal, but not significant loss of model fit [Likelihood-ratio-test: Δχ^2^(4) = 9.12, *p* = 0.058]. The ΔCFI = 0.005 was smaller than the recommended 0.01 cutoff criterion (Cheung and Rensvold, 2002), thus representing negligible loss of fit and supporting the invariance of the factor loadings between the two samples.

Imposing additional constraints referring to the equality of the regression paths in Model 3 also resulted in a small, but insignificant loss of model fit [Δχ^2^(3) = 6.32, *p* = 0.097] with the ΔCFI = 0.004 again not exceeding the cutoff criterion for nested model comparisons. Model 3 showed very satisfying model fit indexes (CFI = 0.984; RMSEA = 0.049) and, despite the imposed constraints, the indirect effect of subjective loss of control on risk-taking behavior via anger was also significant in this model (standardized indirect effects: Germany: 0.10, China: 0.06, both *p*s = 0.003, one-tailed). With the invariance of both the factor loadings and regression paths between the German and Chinese sample and the significant indirect effects, these results provided evidence for the cross-cultural generalizability and supported Hypothesis 4.

### Discussion

Based on the results showing the experimental design’s applicability for inducing externally attributed subjective loss of control in China, and thus in a sample with a different cultural background, this study demonstrated the cross-cultural generalizability of the results found in Main Experimental Study 1. Based on excellent model fits for the mediation path model and the significant indirect effect of anger, the effects of externally caused subjective loss of control on increased risk taking due to the elicitation of anger were replicated in the Chinese sample. Furthermore, multiple group analyses indicated invariance of both the structural and measurement model between the German and Chinese samples. Thus, by comparing the results gained in the Western European and the East Asian sample, this study provided evidence for the hypothesized cross-cultural generalizability of the proposed relationships and the mediating effect of anger.

## General Discussion

These studies investigated how loss of control experiences due to changes in our external environment impact subsequent risk-taking behavior, as well as the role that anger plays in this relationship and the cross-cultural generalizability of the functional mechanisms. In line with the assumptions, loss of control experiences considerably influenced both emotional experiences and subsequent behavior and their impact was demonstrated cross-culturally. Strong support for the hypothesized effect of subjective loss of control experiences on subsequent risk-taking behavior was provided in this set of studies. Subjective control due to changes in external conditions – and thus externally attributable – was shown to arouse anger. Furthermore, anger mediated the relationship between control experiences and risky decision making. These functional relationships and their underlying mechanism were detected in both samples from Western Europe and East Asia and therefore provided support for cross-cultural generalizability. Thus, all of the studies’ results were in line with our hypotheses and contribute to a deeper understanding of how immediately preceding experiences of control impact risk-related decision making in a subsequent, objectively unrelated setting.

As expected, and regarding their close association, the relevance of control experiences for decision making under risk conditions was demonstrated. The decisions of participants exposed to loss of control differed significantly from the decisions of participants who were able to maintain their personal control. More precisely, people who had experienced a gradual loss of control over their outcomes tended to act more riskily in the subsequent decision-making context. This finding might appear rather surprising as prior experiences of loss of control might be assumed to elicit cautious behavior and efforts to regain control instead of giving rise to even more uncertainty. Thus, at first glance, careful and risk-averse behavior might appear to be more plausible and reasonable. However, when reviewing previous research on the effects of chronic losses, wide support for increasing investment can be found (for example in the form of the sunk cost effect, Arkes and Blumer, 1985, and as an escalation of commitment, Staw, 1976). The specific effects of prior loss of control experiences depend on the evaluation of these experiences, for example, the appraisals and attributions of the causes. As supported in the Pilot Experimental Study, the loss of control experience in this study was characterized by an external causation and blockage of one’s goal attainment, but without personally threatening potential. Thus, the emotion of anger aroused by these experiences and its behavioral consequences was the critical aspect predicting subsequent risk taking. As expected, the mediating role of anger was most influential with respect to the increasing risk-taking behavior following the loss of control experiences.

Our results are in line with both theoretical models explaining how emotions impact decision-making processes in general and previous research on the effects of anger in particular. This study adds empirical evidence on the effect of anger on decision making in risky settings. Anger following from subjective loss of control experiences due to changes in external conditions caused increased risk-taking behavior. This observation is compatible with the notion of anger making people feel more confident and encouraging them to actively approach a situation (e.g., see Lerner and Tiedens, 2006).

According to the ATF (Lerner and Keltner, 2000, 2001) the risk-related influence of anger can be explained by high levels of certainty and control appraisals. This aspect is especially interesting in the context of our study in which anger emerged as a powerful mediator of the relationship between loss of control experiences and risk-taking behavior. On the one hand, it provides a feasible explanation for the observed increase in risk-taking behavior. On the other hand, these feelings of high certainty and control may appear counterintuitive and seem to stand in contrast to the subjective loss of control experiences intended to be induced by the experimental manipulation. However, the findings of the Pilot Experimental Study can rule out any doubts that the experimental manipulation to decrease objectively given control may have failed to induce the intended sense of loss of control. In both samples the EG members rated their personal control significantly lower following the experimental manipulation as compared to the CG (and the baseline part). Instead, the apparent “regain” of a sense of certainty and control should be due to the cognitive evaluations of the loss of control experiences, which were shown to be characterized by external attributions and increasing anger, as previously proposed. The emotional experience of anger thus appears to be the crucial underlying mechanism linking the loss of control experiences with the increased risk-taking behavior in the subsequent, objectively unrelated decision-making context. Although elicited by subjective loss of control experiences, anger might have led to a sense of regained certainty due to its accompanying appraisal tendencies and thus has been demonstrated to play a vital role in the context of control and risk. Therefore, the experimental manipulation not only induced subjective loss of control, but – via attribution processes – elicited anger which in turn (via high certainty and control appraisals) impacted the subsequent risk-taking behavior by opposing excessive risk aversion.

Therefore, this study provides support for the influence of emotions as a rather subjective variable influencing the decision-making process. In this study concrete behavior, namely actual risk taking, differed remarkably depending on prior control and emotional experiences. This is in contrast to much other previous research examining possible impacts on decision making, which has focused on perceptions or cognitive estimations of risk instead of actual behavior (e.g., Lerner et al., 2003; Fischhoff et al., 2005). It can be informative to look at precursors of decisions, such as cognitive evaluations of different alternatives, while researching decision making, but it is not sufficient. In the end, the actual choice, the behavior, represents the final outcome of the decision-making process. Thus, by supporting the hypotheses our results both add evidence for the theoretical assumptions and go beyond existing findings by demonstrating effects on behavior.

Furthermore, by exploring the cross-cultural generalizability this study contributed to understanding cultural issues in the relation between control experiences, anger, and risk-taking behavior. Through the comparison of the results of both our samples from Germany, representing Western Europe, and China, representing an East Asian cultural background, the cross-cultural applicability of the proposed relations between the variables was supported. In both Germany and China participants reported significantly higher levels of anger following the externally attributed loss of control and tended to make riskier decisions with this behavioral effect being mediated by anger. In regard to possible cultural influences on our included variables, our findings are remarkably interesting. When focusing on the levels of anger participants reported, one of the variables especially informed by culture, there do in fact seem to be differences between the two samples, with the Chinese anger ratings generally being lower than the German ratings. This is in line with previous findings showing that individualistic and collectivistic cultures generally differ in intensities of reported emotions with China showing particularly low norms and intensity scores (Eid and Diener, 2001). For negative emotions that might threaten the desired interdependence between individuals in collectivistic cultures (Markus and Kitayama, 1991), such as anger, this might be especially true.

It is also worthwhile to have a closer look at the direct effects of the experimental manipulation of personal control and their cross-cultural influences. Despite some evidence for the fact that people from various cultures might differ in their habitually perceived locus of control – with Western European countries tending to perceive higher levels of personal, thus internal, control than Eastern Asian countries – the effects of experiences of loss of control was assumed to be cross-culturally generalizable. In fact, the Experimental Pilot Study showed the effects of the experimental loss of control manipulation on control ratings were exactly as intended in the German and the Chinese sample.

Both the patterns of control ratings and attributions demonstrated the paradigm’s adequacy to induce subjective loss of control attributed to external causes in the Chinese sample as well. These findings counter the argument that the experimental manipulation might have had weaker effects in the Chinese sample since they may be used to lower levels of personal control due to a habitually more external locus of control (see also Scherer and Brosch, 2009, on the relationship of attributions and perceived control) and instead speaks for the cross-cultural applicability of the experimental paradigm. Furthermore, the relevance of experiences and appraisals of personal control for emotions and behavior has been confirmed cross-culturally. The effects of loss of control experiences on risk-taking behavior via anger were also demonstrated in both samples. Additionally, this study supports the applicability of the emotions-behavior link as proposed by the ATF which had previously been questioned for people from collectivistic cultures (Lerner and Keltner, 2001). The effect of anger on risk-related decisions within the Chinese sample was in line with the same direction of the effect in the German sample. In conclusion, despite (possibly habitual) differences in anger ratings and external attributions, the functional relationships between the variables following the loss of control experiences were shown to be generalizable across cultures as hypothesized. One reason for similar mechanisms in both samples might be, that collectivistic and individualistic cultures have more and more converged in recent years due to the economic growing (Hamamura, 2012; Steele and Lynch, 2013).

## Limitations

Although, this study supports all hypothesized functional relationships between subjective loss of control, anger, and risk-taking behavior, and it confirms these findings cross-culturally, there are some limitations that should be taken into account.

The first limitation relates to the limited cross-cultural scope. This study deals with the cross-cultural generalizability of the proposed functional mechanisms. For this purpose, the study was conducted with participants from two countries representing different cultural backgrounds: A sample of German participants representing the Western European culture and a sample of Chinese participants representing the Eastern Asian culture. These cultures are known to differ on a variety of dimensions, among which the differentiation between individualism and collectivism is probably most prominent (e.g., see Triandis, 1994, 1995; Hofstede, 2001). A great deal of research on cultural effects and cross-cultural comparability has been made on the basis of this dimension. Thus, comparing samples differing on this dimension in order to explore cultural influences might be reasonable. Still, one has to keep in mind that drawing final conclusions about cross-cultural universality is – for several reasons – not possible by investigating samples from only two countries. Therefore, until several further replications have been conducted the results of this first step should be handled with care.

However, there are several lines of evidence from the current study that explicitly speak to the interpretability of the reported findings. First, by investigating samples from Germany and China, two countries which are well-known to differ remarkably in a range of aspects, both with respect to country and individual level variables, were chosen. Although differences in mean levels of the variables of interest, such as control perceptions and emotions, are reported in the literature and correspondingly could also be found in our study, the functional relationships were consistent between the two samples, which are in line with the expectations and speaks for their cross-cultural generalizability. Second, in order to evaluate the generalizability of the results, the representativeness of the chosen countries with respect to their cultural area should be considered. For example, China’s individualism score is remarkably lower than the average score of Eastern Asian countries (Hofstede, 2001). From this perspective, the results of this study might even be underestimated, as the difference between Germany and China might be even bigger than the difference between Germany and East Asian countries on average. This deliberation argues strongly for the generalizability between the two cultures. Thus, by comparing a German and a Chinese sample, two samples which can be considered to represent the opposite poles of the often-investigated individualism-collectivism dimension, a first valuable step toward interpreting a cross-cultural generalizability has been made.

The second limitation refers to the context of the experimental induction of loss of control. Our experimental manipulation of changes in objectively given control due to external circumstances was specifically intended to induce a sense of loss of control that was attributable externally, lacked a personal threat, and aroused anger. Through this experimental manipulation, a rather moderate intensity of anger with mean anger ratings around (Germany) or even below (China) the center of the five-point-rating scale was aroused. However, several similar, but not identical scenarios of loss of control experiences are imaginable. For example, slightly different conditions might elicit much stronger intensities of anger that would probably be associated with significant riskier decision making. Furthermore, instead of a continuous change in objective control as applied in our experimental paradigm by decreasing the objectively given control over the course of four rounds of the computer game, the loss of control experiences might have occurred more abruptly and might have aroused surprise rather than anger. Additionally, the changes might have been associated with a feeling of personal threat and thus elicited fear. Therefore, although these alternatives also deal with externally attributable subjective loss of control experiences and would require only small changes in the experimental manipulation that can easily be implemented in our experimental paradigm (e.g., by reducing the number of rounds in the manipulation section or changing financial incentives), their effects on emotions, and subsequent risk-taking behavior are presumed to differ fundamentally. When interpreting our findings it is important to keep the special circumstances of our study in mind, that is, with risk-related behavior impacted by loss of control experiences arousing moderate levels of anger.

## Implications

The results of this study on the hypothesized effect of subjective loss of control on anger and risk-taking behavior suggest several theoretical, methodological, and practical implications as well as raise some questions for future research.

Anger was demonstrated to mediate the relationship between loss of control experiences and increased risk-taking behavior. As proposed by the ATF (Lerner and Keltner, 2000, 2001) the risk-related influence of anger is due to high certainty and control appraisals, which generally accompany and typically characterize this emotion. In our context, aimed at investigating the consequences of subjective loss of control, this contrasting effect appears to be especially interesting. Although in the present study certainty evaluations with respect to the risk-related decision have not been explicitly measured, this aspect deserves more attention and should be addressed in future research. The question arises as to whether experiencing anger could show a way of attenuating the often reported negative effects following subjective loss of control experiences and partly compensates for these consequences, at least in the face of risky decision making. Our study’s results seem to suggest this interpretation, as anger appeared to make people less risk averse. Furthermore, one might suppose this encouraging effect of anger on increasing risk-taking behavior to be especially beneficial when following loss of control experiences since these experiences may hinder bold behavior. Thus, though opposite in nature, the combination of anger and loss of control experiences may contribute to a more risk-neutral decision, as long as the anger is not in excess. Before being able to make more detailed statements, a deeper understanding of the relationship between the obviously contrasting effects of loss of control experiences and certainty experiences due to anger is required. Conditions and limitations of this relationship need to be further investigated, in order to – for example – provide practical implications, such as giving advice for adaptive coping strategies after perceived lack of control in economic or achievement situations.

Related to the question of how loss of control experiences can exert spillover effects to subsequent objectively unrelated settings via emotions is the question about the limits of this mechanism. For example, can purposively elicited or suppressed anger account for adaptive responses either following subjective loss of control or in the face of risk-related decisions? As suggested by Lerner and Keltner (2001, p. 156), awareness is one of the boundary conditions since “becoming aware of one’s own judgment and choice process should deactivate appraisal tendencies, even if the emotion itself persists.” In our experimental design, emotions were assessed via self-report immediately before the decision process. Thus, in both our German and Chinese sample, reflecting on one’s own emotions did not seem to impair the effect of anger on risk-taking behavior. Considering the proposition by Lerner and Keltner (2001), a possible explanation might imply that although the emotion has been assessed via self-report – and thus it is assumed that one is aware of it – it is not cognitively associated with the subsequent decision-making process. In this case it can exert carry over effects between unrelated settings despite awareness of the feeling, which is an interesting and possibly influential assumption certainly worthy of further investigation.

Finally, further research is encouraged as a consequence of the methodological contribution of this study. During the Pilot Experimental Studies, an experimental paradigm for inducing externally attributable subjective loss of control (and consequently anger) was developed. Its adequacy was successfully supported in both the German and the Chinese sample, thus additionally suggesting its cross-cultural applicability. This methodological innovation facilitates further research on the effects of loss of control experiences due to external causes, an issue that up to now largely lacked proper methods for investigation. Considering people’s everyday experiences of externally caused loss of control, the newly developed and herein introduced experimental paradigm provides easy access to this highly relevant research topic.

## Conclusion

Perceiving oneself to be a competent person and in control of personally relevant outcomes has generally been proposed to be a fundamental human need (Skinner, 1996). Therefore, the importance of control perceptions for emotions and behavior has already been a focus of research for quite a time, often with a concentration on the detrimental consequences of lack of control experiences. However, in the face of ongoing technological and societal developments that may make feelings of lacking control and uncertainty an everyday experience, this issue has not lost its relevance. Instead, questions of how to adaptively cope with experiences of lack of control and uncertainty, how to encounter their consequences, and the mechanisms of their impact on subsequent decision-making processes are quite timely. By applying a cross-cultural perspective, the understanding of fundamental general functional relationships as well as cultural influences on our everyday coping mechanisms in response to universal challenges is fostered.

This study’s results point out that – across cultures – emotions, more precisely, anger, might bridge the gap between prior, subjective loss of control experiences and the subsequent decision-making setting, and thus can account for prior proven spillover effects. Furthermore, despite the often rather negative evaluation of anger as a socially undesirable and unpleasant emotion, our findings may give reason to cast a more positive light on it; anger can also be associated with cognitions of power, control, and certainty. It seems to be able to compensate for the discouraging effects of lack of control experiences by counteracting overly cautious behavior and contributing to more confident, optimistic and risk-taking decision-making. Intensified efforts aimed at getting to the bottom of the underlying mechanisms might shed further light on the theoretical and practical opportunities and constraints of these seemingly promising effects of anger, which appear to have the potential to support people in coping with experiences of lacking personal control and uncertainty.

## Conflict of Interest Statement

The authors declare that the research was conducted in the absence of any commercial or financial relationships that could be construed as a potential conflict of interest.

## Footnotes

1 There were no significant differences (all *p*s > 0.05) between the treatment groups with respect to the socio-demographic variables, non-verbal reasoning indicators, and general control beliefs, thus supporting the treatment groups’ equivalence.

2 The *t*-tests for equivalence did not reveal any significant group differences regarding the socio-demographic and trait variables (all *p*s > 0.05).

3 Our experimental manipulation was designed to induce specifically the emotion of anger (for the development of this manipulation, see Wimmer et al., 2009). In order to be able to judge whether our manipulation would indeed have first of all effects on anger experiences, we measured anger as well as nine other emotions (anxiety, fear, sadness, shame, hopelessness, boredom, enjoyment, pride, and hope) as a manipulation check after t2. In line with our expectation, the change of the level of anger from t1 to t2 in the experimental group (EG) was significantly higher than those of nine other emotions (a repeated measures MANOVA, pairwise comparisons from the emotion (t1/t2) × treatment condition (EG/CG) interaction revealed all *p*s < 0.001). Thus anger was the emotion which was by far most strongly triggered by the manipulation.

4 Before replicating the main experimental study, a pretest similar to the German pilot study was applied in order to test the design’s adequacy to induce externally attributed subjective loss of control in Chinese participants. The sample consisted of *N* = 63 (68% female) participants (age: *M* = 21.10 years, *SD* = 1.84, range: 18–26) with random assignment of *n* = 32 (69% female) participants to the EG and *n* = 31 (68% female) participants to the control group (CG). *t*-tests for equivalence showed no significant group differences (all *p*s > 0.05) between the EG and CG with respect to socio-demographic and trait variables (locus of control: IPC by Levenson, 1972, German version by Krampen, 1981; risk propensity: DOSPERT by Weber et al., 2002, German version by Johnson et al., 2004; Chinese items for both measures had been developed during this study based on the German versions). In line with the expectations, at t1 there were no significant group differences in the ratings of subjective control [CG: *M* = 3.90, *SD* = 1.14; EG: *M* = 3.72, *SD* = 1.28; *t*(61) = -0.61, *p* = 0.547, *d* = -0.15], internal [CG: *M* = 3.94, *SD* = 1.59, EG: *M* = 3.98, *SD* = 1.46; *t*(61) = 0.127, *p* = 0.899, *d* = 0.03] and external attributions [CG: *M* = 1.39, *SD* = 1.14, EG: *M* = 1.81, *SD* = 1.05; *t*(61) = 1.541, *p* = 0.129, *d* = 0.39]. However, following the experimental manipulation at t2, the EG rated their perceived control significantly lower (*M* = 1.14, *SD* = 1.12) than the CG [*M* = 4.08, *SD* = 1.12; *t*(61) = -10.44, *p* < 0.001, *d* = -2.63] and showed significantly higher external attributions (*M* = 4.02, *SD* = 1.88) as compared to the CG [*M* = 1.65, *SD* = 1.22; *t*(61) = 7.82, *p* < 0.001, *d* = 1.97] and also as compared to t1 [*t*(31) = -8.098, *p* < 0.001, *d* = -1.43]. Accordingly, the EG’s internal attributions at t2 were significantly lower (*M* = 1.50, *SD* = 1.11) as compared to the CG’s [*M* = 3.90, *SD* = 1.37; *t*(61) = -7.633, *p* < 0.001, *d* = -1.92] and also as compared to t1 [*t*(31) = 7.915, *p* < 0.001, *d* = 1.41]. Thus, the attributional pattern within the EG following the experimental manipulation was characterized by low internal and high external attributions as intended and expected. These results were comparable to those of the German sample and provided evidence for the applicability of the experimental design to Chinese participants which we considered as a prerequisite for conducting Main Experimental Study 2.
